# Hydrodynamic particle focusing enhanced by femtosecond laser deep grooving at low Reynolds numbers

**DOI:** 10.1038/s41598-021-81190-y

**Published:** 2021-01-18

**Authors:** Tianlong Zhang, Misuzu Namoto, Kazunori Okano, Eri Akita, Norihiro Teranishi, Tao Tang, Dian Anggraini, Yansheng Hao, Yo Tanaka, David Inglis, Yaxiaer Yalikun, Ming Li, Yoichiroh Hosokawa

**Affiliations:** 1 Division of Materials Science, Graduate School of Science and Technology, Nara Institute of Science and Technology, Ikoma, 630-0192 Japan; 2 School of Engineering, Macquarie University, Sydney, 2122 Australia; 3 Center for Biosystems Dynamics Research, RIKEN, Osaka, 565-0871 Japan

**Keywords:** Laser material processing, Lab-on-a-chip

## Abstract

Microfluidic focusing of particles (both synthetic and biological), which enables precise control over the positions of particles in a tightly focused stream, is a prerequisite step for the downstream processing, such as detection, trapping and separation. In this study, we propose a novel hydrodynamic focusing method by taking advantage of open v-shaped microstructures on a glass substrate engraved by femtosecond pulse (fs) laser. The fs laser engraved microstructures were capable of focusing polystyrene particles and live cells in rectangular microchannels at relatively low Reynolds numbers (Re). Numerical simulations were performed to explain the mechanisms of particle focusing and experiments were carried out to investigate the effects of groove depth, groove number and flow rate on the performance of the groove-embedded microchannel for particle focusing. We found out that 10-µm polystyrene particles are directed toward the channel center under the effects of the groove-induced secondary flows in low-Re flows, e.g. Re < 1. Moreover, we achieved continuous focusing of live cells with different sizes ranging from 10 to 15 µm, i.e. human T-cell lymphoma Jurkat cells, rat adrenal pheochromocytoma PC12 cells and dog kidney MDCK cells. The glass grooves fabricated by fs laser are expected to be integrated with on-chip detection components, such as contact imaging and fluorescence lifetime-resolved imaging, for various biological and biomedical applications, where particle focusing at a relatively low flow rate is desirable.

## Introduction

Microfluidic technology has been increasingly used to isolate and analyze micro-scale particles for a wide range of applications in the fields of biology, biomedicine and environment, due to its various advantages, such as low sample and reagent consumption, improved sensitivity and efficiency, and reduced analysis time^1–4^. Particle focusing by microfluidics, which allows the control of particle positions in a tightly confined flow stream in microchannels, is a necessary step prior to detection, trapping and separation at the downstream^5–8^. For example, flow cytometry employs two sheath fluids to focus cells for the detection and measurements of physical and chemical characteristics of cells^9^. Moreover, particle focusing has been integrated with other on-chip functional components in microfluidic devices for the manipulation and analysis of different types of synthetic and biological particles, such as micelles, bacteria, microalgae, normal and cancer cells^10–12^.

A variety of microfluidic techniques have been developed for the focusing of particles, which can be categorized into two groups: active and passive techniques^13^. Active techniques, such as thermophoresis^14^, dielectrophoresis (DEP)^15^, optical trapping^16^ and acoustophoresis^17^_,_ apply external forces to achieve particle focusing in microchannels. However, these techniques require complicated fabrication processes or bulky external setup, and the external actuation may cause negative effects (e.g. cell damage by heating or cavitation)^18^. On the other hand, passive techniques, such as deterministic lateral displacement (DLD), hydrophoresis, inertial and viscoelastic focusing are based on internal channel, microstructure topology or medium properties to generate hydrodynamic forces to direct particles^13,19^_,_ which feature simplicity in experimental setup. DLD uses pillar arrays to focus and deflect particles having a similar size to the pillar gap^20^, but it is limited by issues of pillar clogging and anisotropic permeability, is complicated and expensive to fabricate, and only focuses in two dimensions^21^. Inertial focusing usually works at intermediate Reynolds numbers (Re) typically ranging from 1 to 100^22^_,_ and the inertial effects become increasingly insignificant when Re goes down to 1^23^. However, the focusing of particles at low Re, e.g. physiological flow at speeds less than 1 mm/s^24^, in microchannels is still of interest to be explored, as it plays an important role in image-based detection, such as contact imaging^25^ and fluorescence lifetime-resolved imaging^26^. Although viscoelastic focusing is capable of aligning microparticles at Re less than 1, it requires to carefully tune the rheology properties of suspending medium containing polymers, such as poly-ethylene-oxide (PEO)^27^, polyvinyl-pyrrolidone (PVP)^28^, hyaluronic acid (HA)^29^ and DNA^30^. By now, researchers have demonstrated the feasibility of particle focusing at Re less than 1 by hydrophoresis in microchannels patterned with microstructure arrays^31^. Hsu et al*.* achieved the focusing of 9.9-µm polystyrene particles using multiple sets of herringbone structures when Re is 0.012^32^. Conventionally, photolithography and wet etching are used for the fabrication^31,33^, but they need a long time to execute deep etching to create microstructures with the use of chemical reagents for effective particle focusing. Therefore, it calls for rapid and simple techniques that can create microstructures in microchannels for particle focusing and manipulation.

Femtosecond pulse (fs) laser is a promising tool for the fabrication of microfluidic devices made from glass^34,35^. Glass is optically transparent, mechanically and thermally stable, electrically insulating, and solvent compatible, thus making it attractive as a substrate material for microfluidic devices^36^. Tight focusing of ultrashort (~ 100 fs) infrared laser pulses of moderate energy (typically ranging from 1 to 100 µJ) into glass can result in high localized intensities (in excess of 10^14^ W/cm^2^)^37^. At the laser focal point, the laser pulses are absorbed by electrons in the glass through multiphoton ionization, leaving behind a localized permanent ablation of the material^38,39^. The fs laser micromachining shows advantages over other methods such as wet etching^40^ and deep reactive ion etching (DRIE)^41^ for rapid and direct deep writing in glasses^42–44^, enabling the fabrication of different types of glass-based microfluidic devices, such as a 12-μm ultra-flexible chip^45^ and a high-throughput microparticle filter^46^.

In this study, we achieved continuous hydrodynamic focusing of polystyrene particles and live cells with different sizes using fs laser engraved grooves in a rectangular microchannel. Numerical simulations were performed to explain the mechanisms of particle focusing, which is based on local microstructure-induced secondary flows. Besides, we demonstrated that the groove-induced hydrodynamic forces have an accumulative effect on the particle lateral displacement. We also systematically studied how the factors, i.e. groove number, groove depth and flow rate affect particle focusing in microchannels embedded with fs laser engraved groove arrays. To the best of our knowledge, it is the first report on fs laser engraved glass surficial microstructures for particle focusing in microfluidics. The deep grooving realized by the fs laser processing allows effective particle focusing at Re less than 1.

## Results

### Glass groove microstructures by femtosecond pulse laser

**Figure 1:**
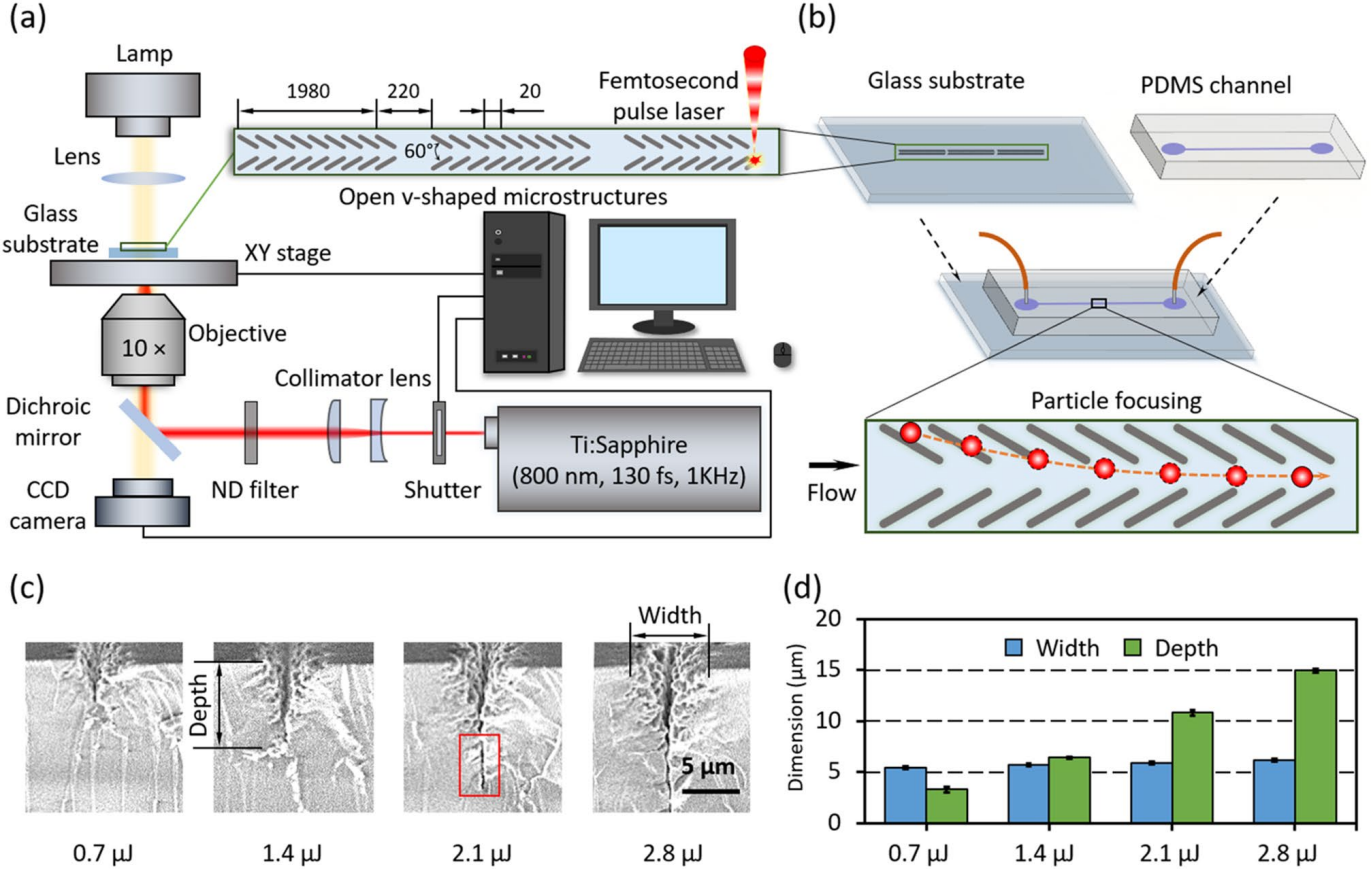
Femtosecond pulse laser engraved glass surficial microstructures for particle focusing in a microchannel. (**a**) Schematic illustration of the laser engraving system. (**b**) Schematic illustration of the microfluidic device for continuous focusing of microparticles. (**c**) SEM images of top and bird’s-eye views of the grooves engraved by four different pulse energies: 0.7, 1.4, 2.1 and 2.8 µJ/pulse. (**d**) Plots of width and depth of grooves against the engraving pulse energy.

Arrays of open v-shaped microstructures were patterned on glass substrates (Borosilicate, 76 × 26 × 1t, Matsunami Glass ind., Ltd., Video S1) by a near infrared (NIR) fs laser for particle focusing (Fig. 1a, b). SEM images of grooves engraved under different pulse energies were obtained and compared (Fig. 1c). The slit-like structure highlighted by the red box was observed. Results showed that the groove widths are 5.4 ± 0.2, 5.7 ± 0.1, 5.9 ± 0.3 and 6.2 ± 0.2 µm, and the groove depths are 3.3 ± 0.2, 6.4 ± 0.2, 10.8 ± 0.2 and 15.0 ± 0.4 µm, respectively, when the pulse energies are 0.7, 1.4, 2.1 and 2.8 µJ/pulse, respectively (Fig. 1d). The SEM analysis revealed that the grooves with similar width were formed when the energy of fs laser increases from 0.7 to 2.8 µJ/pulse, while the depth of the grooves is strongly dependent on the laser energy.

**Figure 2:**
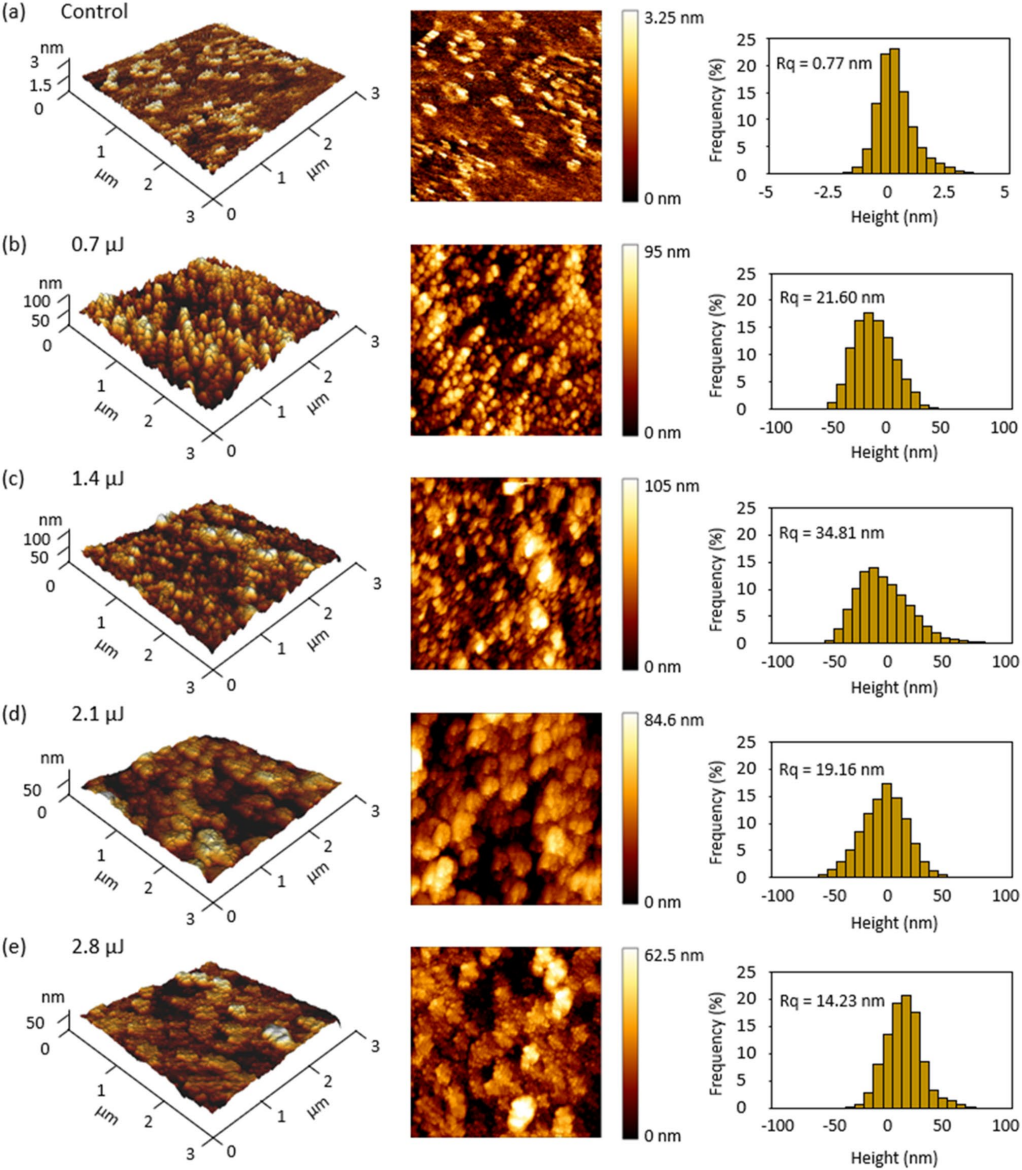
Surficial roughness detection by atomic force microscopy (AFM). (**a**) Control group. (**b**–**e**) Surrounding areas of the grooves engraved under pulse energies of (**b**) 0.7, (**c**) 1.4, (**d**) 2.1 and (**e**) 2.8 μJ/pulse. (We used the software JPK data processing 6.1 to create the image).

Surface morphology near the grooves was detected by atomic force microscopy (AFM) and evaluated as root-mean-square (RMS) roughness (Rq). The groove width was almost consistent with the data obtained from SEM analysis [Fig. S1(a-c)], although the depth was unclear by the AFM analysis because of the probe height limitation of 15 μm and the difficulties in detecting the slit-like structures (Fig. 1c red box). Small debris was observed near the edge of grooves on the glass surface (Fig. 2), causing Rq value in the laser engraved groups tens of nanometers higher than that in the control group [Fig. S1(d)]. Histogram analysis showed that the height values in control group mainly range from -2.5 to 2.5 nm (Fig. 2a) while the values in the groove surrounding areas mainly range from -50 to 50 nm (Fig. 2b–e). If the particles sink and flow on the surface of the channel bottom, mechanical interactions between particles and the groove microstructures would affect the particle motion. AFM results showed that the debris is at nanometer scale thus is not big enough to block the micrometer sized particles used in this study to further change their movement directions. Besides, roughness is almost not dependent on the laser energy for the laser engraved groups and mainly ranges between 15 to 35 nm, denoting similar friction influences due to the roughness.

**Figure 3:**
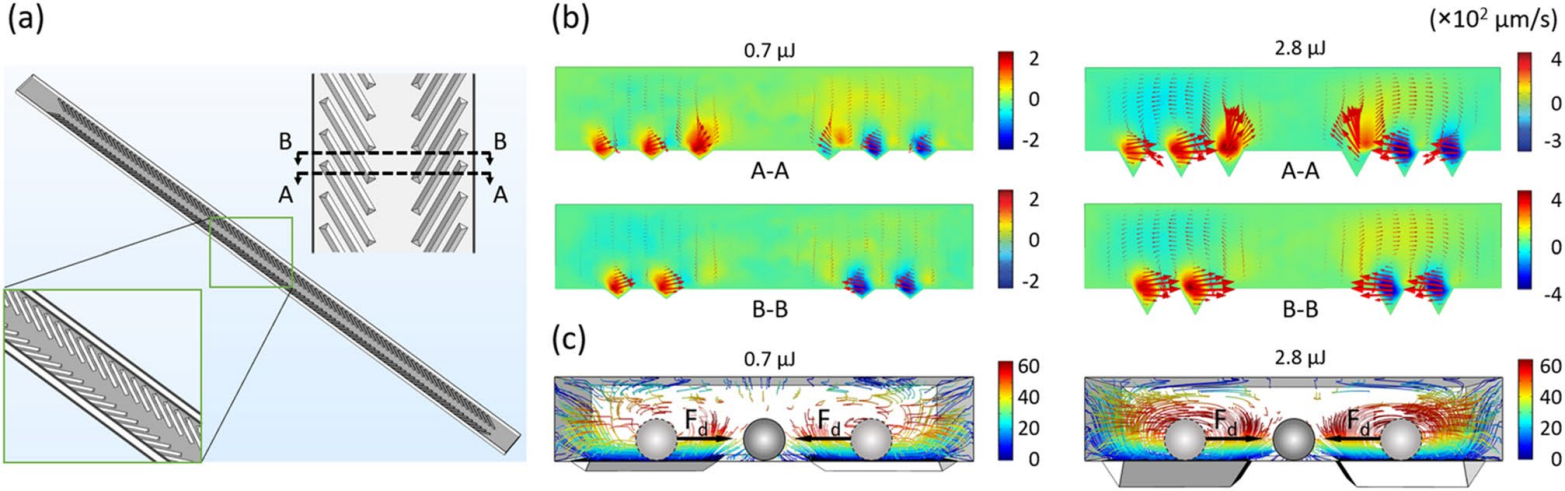
Numerical analysis of flow fields for particle focusing in a microchannel with fs laser patterned grooves. (**a**) 3D structure of the microchannel used in the simulation. The insets are enlarged views of the grooves. (**b**) 2D vector plots of flow velocity fields at cross-sections of the microchannel. Red arrows are scalars shown in a proportional way. (**c**) Front view of the channel with 3D flow streamlines with schematic illustrations of expected particle lateral displacements according to (**b**) for non-buoyant particles. (We used the software COMSOL Multiphysics 5.4 to create the image).

Further, we performed numerical simulations to investigate the fluid behaviors induced by the groove microstructures. We considered the groove depth dependence of the fluid behaviors. Based on the groove structures in SEM images, simplified three-dimensional (3D) models containing one block of groove structures were built in COMSOL Multiphysics software (Fig. 3a) for the visualization of flow streamlines at Re = 0.13 to evaluate the hydrodynamic forces acting on the particles^47–49^. The depth of the slit-like groove highlighted by the red box was excluded from the model (Fig. 1c). Two typical channel cross-sections having either six or four grooves were shown with the distribution of two-dimensional (2D) velocity fields (Fig. 3a, b)^50^. We found that the groove-induced local secondary flows are stronger at the area closer to the surface of the grooves, and the magnitude of flow velocity decreases with the increase in the distance from the grooves [see 1.4- and 2.1-μJ conditions in Fig. S2(a)]. In contrast, no secondary flows were observed in the control microchannel [Fig. S2(b)]. We also found out that the helical secondary flows were enhanced mainly by the increase in groove depth. For example, the 2D secondary helical flows induced by grooves achieved by 2.8 μJ/pulse fs laser were almost 2 times stronger than that of grooves achieved by 0.7 μJ/pulse fs laser (Fig. 2b), resulting in, therefore, the stronger 3D helical flows in the microchannel with deeper grooves (Fig. 3c). The groove microstructures in a microchannel induce pairs of local helical secondary flows (net lateral motion of fluid), which are able to direct particles at the bottom of the channel to the equilibrium positions at the center of the channel due to viscous drag^51^. The particles experience not only a force toward the center of the channel but also upward, that pushes the particles toward the upper part of the microchannel (Fig. 3b). Although the upward force may produce an equilibrium position for the particles, groove periodic appearances are likely to make the position unstable for the particles above in a 3D environment.

When Re is far less than 1, viscous effects dominate over inertial effects^52,53^. Particle motion is determined by the viscous effect of the suspending medium based on Stokes’ law^54,55^. The hydrodynamic drag force applied on a particle can be defined as *F*_*d*_ = 6*πηRv*, where *η* is medium dynamic viscosity, *R* is the radius of the spherical particle, and *v* is the relative velocity between particle and medium^56^. At a low flow speed (low Re), particles sink and flow close to the bottom of the microchannel where groove arrays are patterned. Since the fluids over the patterns at the bottom of the channel move from the channel sidewalls to the channel center, the generated Stokes’ drag forces direct particles in that region toward the equilibrium positions at the channel center.

### Effect of groove number

**Figure 4:**
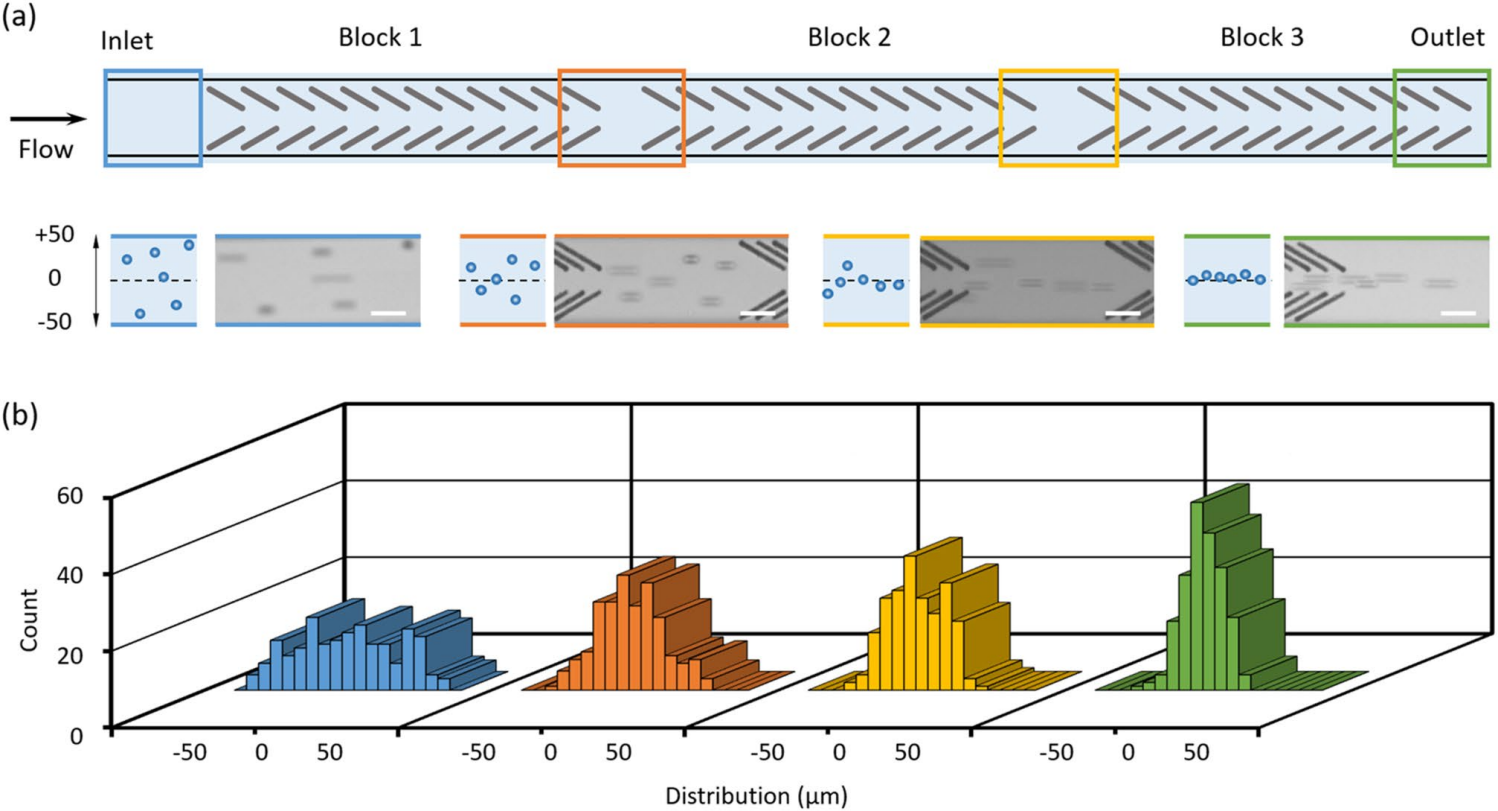
Effect of open v-shaped patterns on particle focusing. (**a**) Schematic illustration of a microfluidic channel having three blocks of groove arrays. Schematics and superimposed experimental images depicting the distributions of polystyrene particles at the inlet, after passing block1, 2 and 3 (outlet) are shown. Scale bar is 40 µm. (**b**) Plots of the lateral distributions of particles along channel width. The length of one block is 1980 µm and the distance between neighboring two blocks is 220 µm. The distributions of particles at four different locations are recorded and compared: inlet (before passing block 1), after passing block 1, 2, and 3 (outlet). 200 particles are measured for each group.

A microchannel having three consecutive blocks engraved at a pulse energy of 2.8 μJ/pulse was used to investigate the influence of groove number on particle focusing (Fig. 4a). Solutions of 10-µm polystyrene particles were injected into the microchannel at 450 nL/min (Re = 0.13). Experimental results showed that the polystyrene particles were able to be focused to a narrow stream at the channel center in the microchannel patterned with groove arrays (Fig. 4b), but no focusing effect was observed in the control channel (plain microchannel without any patterns, see Fig. S3). Particles randomly distributed at the inlet (0.0 ± 22.4 µm) were gradually directed toward the channel center after passing through all the blocks (Fig. 4b), and 84% particles were focused within the 22-µm wide stream at the outlet (0.3 ± 8.4 µm). It showed that the focusing performance was gradually enhanced with the increase in the number of the grooves when particles pass through, thus groove- induced hydrodynamic forces have an accumulative effect on the particle lateral displacement.

### Effects of groove depth

**Figure 5:**
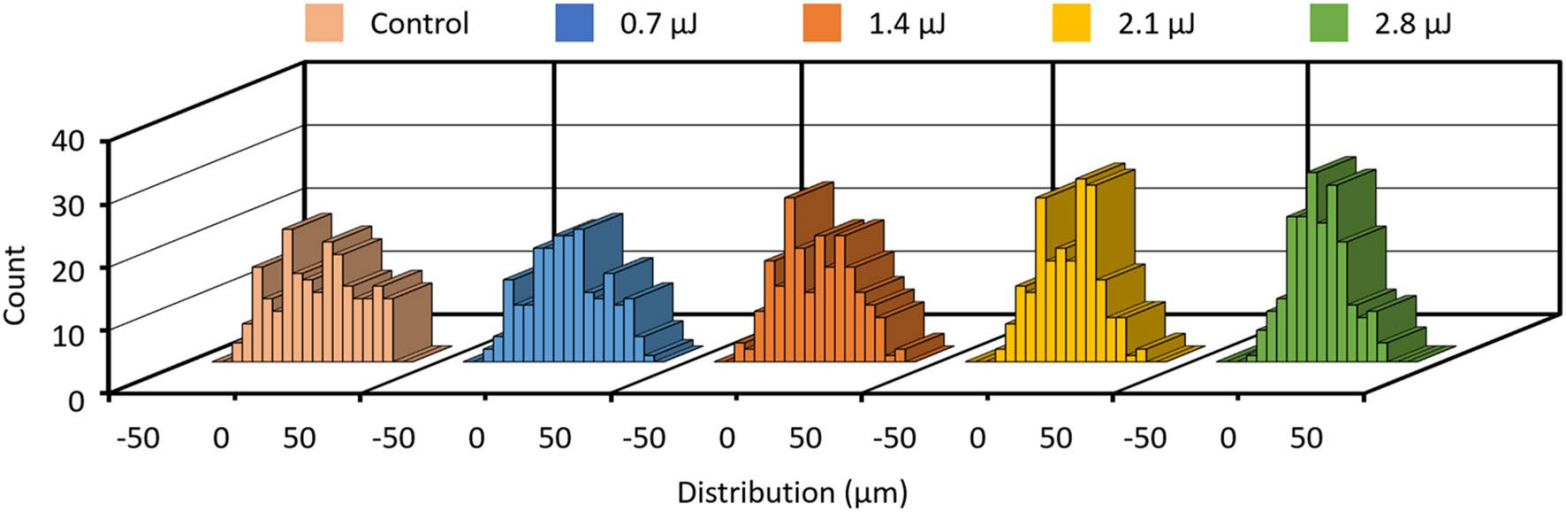
The performance of focusing 10-µm polystyrene particles using the open v-shaped microstructures achieved by femtosecond pulse laser at different energies of 0 (control), 0.7, 1.4, 2.1 and 2.8 µJ/pulse.

We further investigate the effect of groove depth on the performance of groove-patterned microchannels for particle focusing. Solutions of 10-μm polystyrene particles were injected into the channels having grooves of different depths achieved by fs laser at different energies at a flow rate of 450 nL/min (Re = 0.13). We found out that the focusing performance at the end of the first block was enhanced with the increase in the energy-dependent groove depth (Fig. 5). The distribution of particle lateral positions across channel width at the end of the first block became increasingly narrow with the increase of laser energy: 0.4 ± 19.5 μm (0.7 µJ), -0.2 ± 18.6 μm (1.4 µJ), -0.1 ± 16.0 (2.1 µJ) to 0.1 ± 14.2 μm (2.8 µJ). In contrast, we failed to find any focusing phenomenon in the control microchannel without grooves, as the average distribution of particle lateral positions in the channels were -1.0 ± 22.3 μm (at inlet) and 0.2 ± 21.6 μm (at outlet).

These results demonstrated the hydrodynamic particle focusing is enhanced with the increase of depth of the grooves enabled by femtosecond laser deep grooving. Further, particles flowing in microchannel patterned with the open v-shaped microstructures were recorded under an optical microscope. It was observed that the polystyrene particles were gradually directed toward the channel center in ~ 3 s when the laser energy is 2.8 µJ/pulse (video S2), while the lateral movement toward the channel center was not obvious when the laser energy is 0.7 µJ/pulse (video S3). Then, the microchannel with open v-shaped microstructures engraved at a pulse energy of 2.8 μJ/pulse was used to investigate the effect of flow rate and the focusing of biological samples.

### Effect of flow rate

**Figure 6:**
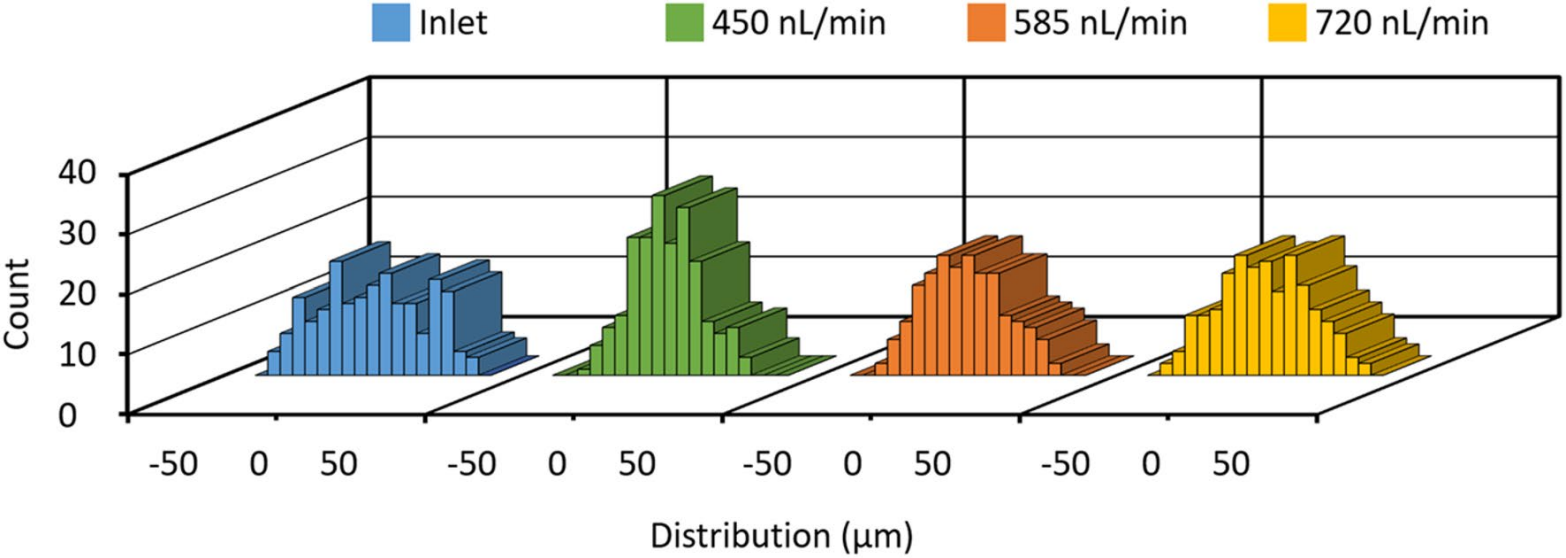
The effect of the flow rate on the focusing of 10-µm polystyrene particles in the microchannel. The injection flow rates are 450, 585 and 720 nL/min.

To explore the effect of flow rate on particle focusing, 10-μm polystyrene particle suspensions were injected into a microchannel with the microstructures at three different flow rates: 450, 585 and 720 nL/min. Re are 0.13, 0.17 and 0.21, respectively. We found that the focusing gradually decreases with the increase in flow rate at the end of the first block (Fig. 6). With flow rate of 450 nL/min (Re = 0.13), 60.5% particles were focused to the middle open space (0.1 ± 14.2 μm). In contrast, 47% and 39% particles were directed to the open space with average lateral positions of -0.1 ± 17.0 μm and 0.5 ± 19.2 μm at the end of the first block, respectively, when the flow rates are 585 nL/min and 720 nL/min, respectively. These results denoted the focusing performance can be promoted by decreasing the flow rate. It is likely because that slow fluid flow rate results in particle sedimentation within the microchannel, thus interacting with the center-moving secondary flows induced by grooves (Fig. 3b, c).

### Hydrodynamic focusing of live cells

**Figure 7:**
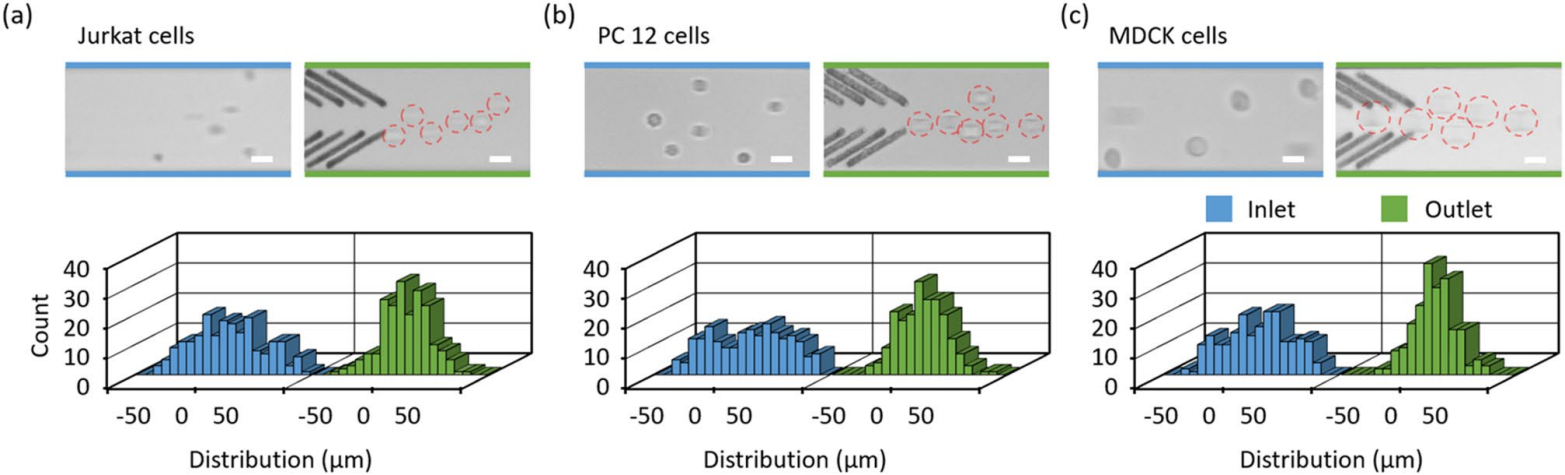
Focusing of live cells by the microfluidic device with fs laser patterned arrays of open v-shaped microstructures. The lateral positions of (**a**) Jurkat cells, (**b**) PC 12 cells and (**c**) MDCK cells at the inlet and outlet are recorded and compared, when the flow rate is 180 nL/min. Superimposed experimental images of cell distribution are presented. Red dotted circles denote cells. Scale bar is 20 µm. 200 cells are measured for each group.

To demonstrate the capability of the microchannel embedded with grooves for the focusing of biological samples, Jurkat, PC 12 and MDCK cells were used. Three different flow rates including 450, 315 and 180 nL/min were used. Re are 0.13, 0.09, 0.05 respectively. We note that cell viability is unlikely to be significantly affected, since the focusing of cells is performed at low Re (< < 1)^57,58^. Almost no significant focusing performance was found at the end of the third block (outlet) for the Jurkat cells when the flow rates were 450 and 315 nL/min [Fig. S4(a)]. However, the focusing phenomenon appeared at the outlet when the flow rate is 180 nL/min, and 60% Jurkat cells were directed to the 22-µm wide stream along the channel center with an average lateral position of -0.1 ± 14.3 μm (Fig. 7a). Particle tracing demonstrated that the Jurkat cell was able to be gradually directed toward the channel center at this flow rate (video S4).

Compared to Jurkat cells, PC 12 cells were able to be focused at 450 nL/min at the outlet [Fig. S4(b)] and the focusing performance was increasingly enhanced with a decrease in flow rate. With a flow rate of 180 nL/min, 53% PC 12 cells were arranged to the 22-µm wide stream along the channel center at outlet with an average lateral position of -0.5 ± 13.9 μm (Fig. 7b). MDCK cells showed a similar focusing performance to PC 12 cells [Fig. S4(c)]. 67% MDCK cells were arranged to the 22-µm wide stream along the channel center with an average lateral position of 1.3 ± 12.5 μm (Fig. 7c), when the flow rate is 180 nL/min (see the tracing of the MDCK cell within the one-block length in video S5). Compared to the focusing of polystyrene particles under the same experimental conditions at outlet (0.3 ± 8.4 µm, Fig. 4b), the performance of the microdevice for the focusing of live cells was slightly decreased. Variations in cell properties, such as cell mass density, shape and size might be accountable for it.

## Discussion

When a glass material is excited by an intense NIR fs laser beam with Gaussian distribution, the material excited by the multiphoton absorption acts as a lens to focus the pulse itself^59,60^. On the present machining condition, e.g. 2.8 µJ/pulse, that the fs laser is focused at the surface through the objective lens, the fs pulse propagates in the glass while keeping the focusing itself and creates a deep groove with a depth over 10 µm in a very short time (Fig. 1c). Since the focusing diameter almost corresponds to the diffraction limit less than 5 μm, the groove has a high aspect ratio. This narrow and deep groove is a unique structure realized by the fs laser machining.

The AFM surface analysis demonstrated that the surface roughness due to fs laser engraving is almost not dependent on the laser energy (Fig. 2), while the focusing performance was promoted with the increase in the energy pulse (Fig. 5). Therefore, the groove induced secondary flows play a dominated role in driving particles laterally toward channel center (Fig. 4). The hydrodynamic force localized closer to the groove microstructures carries the particles like a conveyor belt (Fig. 3). We can arrange routes of the belt flexibly in the microfluidic channel by the fs laser processing. For example, by decreasing the interval distance of the two neighboring grooves, the focusing performance can be promoted (Fig. S5). Also, the noncontact machining of grooves enables to add the belts even after fabricating the microfluidic channel with closed structure.

The device (2.8 µJ/pulse) demonstrates a better ability to arrange particles at relatively lower injection flow rates for both polystyrene particles and cells. For example, the focusing performance is able to be further promoted at 315 nL/min for 10-µm polystyrene particles (Fig. S6). But the particle flow became unstable with injection flow rates down to tens of nanoliters per minute. Similarly, the performance of the groove arrays for focusing 4.5-µm polystyrene particles is enhanced by decreasing the injection flow rate from 450 to 315 nL/min (Fig. S7). It is likely that a higher flow rate induces relatively stronger secondary flows, which may reduce the focusing efficiency. Taking advantage of the size-dependent focusing performance, the groove arrays allow the separation of 4.5- and 10-µm polystyrene particles (Fig. S8). One of our future studies is further optimization of the groove structures to enhance the separation efficiency. Besides, it demonstrates potentials in particle imaging related techniques. Since the focused particles at the outlet are likely to be quite close to the channel bottom, denoting almost similar height positions in the channel (Fig. S9).

## Conclusion

In this study, we patterned groove arrays on glass substrates using fs laser and achieved hydrodynamic focusing of polystyrene particles and live cells in low-Re flows (Re < 1). The focusing performance of patterned microchannels was found to be enhanced by fs laser deep grooving and improved with the increase in groove number. One task of our future work is to optimize the parameters of the device, such as the distance between the two opposite grooves, length of the groove pattern, channel height, groove width, and angle for improvement in the focusing of different types of particles. Also, the ability of the device to arrange particles to the channel center is hoped to be enhanced by decreasing the distance between the two opposite grooves^32^. This device with desirable features, e.g. low requirements for carrier medium preparation, simple device construction and flexible design, shows potentials in the integration with on-chip detection components, e.g. contact imaging and fluorescence lifetime-resolved imaging, for cell profiling. We also expect to develop more capabilities, e.g. particle sorting and trapping, by the further design of groove structures. Although the laser scanning method is better for rapid prototyping to verify the working principle, lithography is better for the mass production. When the application is established in the next step, lithography will be considered to improve the uniformity of the grooves. Besides, it is interesting to further investigate the particle movement behaviors to have a more in-depth understanding of the focusing mechanism, e.g. the role of particle rolling. By integrating with functional on-chip techniques, this approach based on fs laser machining of microstructures on glass substrates is expected to be used for particle focusing and manipulation in various fields of biology, biomedicine and environment.

## Materials and methods

### Design and fabrication of open v-shaped patterns

Laser pulses from a Ti:Sapphire fs laser amplifier (Spectra-Physics, Solstice-Ref-MT5W, 800 nm, 130 fs, 1 kHz) sequentially passed through a shutter, collimator lens and an ND filter (Fig. 1a). Then the pulses were introduced to a 10 × objective lens (NA. = 0.25) equipped on an inverted microscope (Olympus, IX71) and focused on a glass substrate for glass engraving. The diameter of the engraving spot was ~ 2 µm. By controlling a motorized stage (Sigma Koki, E-65GR) with a speed of 100 µm/s, open v-shaped patterns were created. The number of overlapping pulses per position in the fabrication process was 20. Pulse of different energies, i.e. 0.7, 1.4, 2.1 and 2.8 µJ/pulse were used to fabricate open v-shaped microstructures with different depths. On the surface of the glass substrate, 100 pairs of grooves were engraved, which are defined as one block, and three consecutive blocks were formed in one microchannel (Fig. 1a). The interval distance between two grooves was set at 20 µm in the etching programming. The pattern width was ~ 94 µm and the space in the middle was ~ 22 µm wide. The distance between two neighboring blocks was 220 µm, aiming to leave enough space for observation (see Fig. 1a). The engraved glass substrates were immersed into tap water for 10-min ultrasonic cleaning, which is followed with nitrogen drying.

### Fabrication of microfluidic device with open v-shaped micropatterns

A microfluidic device for particle focusing consists of two layers: a polydimethylsiloxane (PDMS) layer having a straight rectangular microchannel, one inlet and one outlet, and a glass substrate with laser engraved grooves (Fig. 1b). The PDMS microfluidic channel of 100 μm in width and 20 μm in height was bonded to the laser engraved glass substrate after plasma treatment (Plasma Cleaner CY-P2L-B). Briefly, the channel embedded in PDMS was partially bonded to the glass substrate to reduce movements between them. Then the irradiated glass and the PDMS channel were manually aligned under an optical microscope carefully. The samples containing polystyrene particles or live cells were infused into the microfluidic device using a 250-μL Hamilton syringe by a syringe pump (Harvard Apparatus 11 Elite). The distributions of lateral positions of particles across microchannel width before and after passing the arrays of grooves were monitored and recorded under an inverted microscope (Axiovert 135, Carl Zeiss, Germany).

### Particle suspension preparation

To evaluate the performance of the microfluidic device for particle focusing, 10-µm polystyrene particles (Polybead, Polysciences, 10.0 ± 1.0 µm, ~ 1.05 g/cm^3^) at an initial concentration of 4.55 × 10^7^ particles/mL and 4.5-µm polystyrene particles (Polybead, Polysciences, 4.5 ± 0.2 µm, ~ 1.05 g/cm^3^) at an initial concentration of 4.99 × 10^8^ particles/mL were used. The particle suspensions were stored at a 4 °C and diluted to 5.0 × 10^5^ particles/mL with pure water just before experiments.

### Cell preparation

Human T-cell lymphoma Jurkat (Jurkat, RCB0537), dog kidney cell (MDCK, RCB0995), and rat adrenal pheochromocytoma (PC 12, RCB0009) were provided by the RIKEN CELL BANK (Tsukuba, Japan). Jurkat and MDCK cells were grown to 70–80% confluence on culture dishes with a diameter of 10 cm in high glucose Dulbecco’s Modified Eagle’s Medium (DMEM, 4.5 g/L glucose) supplemented with fetal bovine serum (FBS, 10%) and antibiotic agents (100 units/ml penicillin, 100 μg/ml streptomycin) under CO_2_ (5%) and saturated water vapor at 37 °C. PC12 cell line was cultured on the dish in low glucose DMEM (1 g/L glucose) with FBS (10%), horse serum (10%), and the antibiotic agents under the same environmental condition. The cells were detached from culture dishes by trypsinization (50 μg/mL trypsin, 53 μM EDTA) followed by suspending in the culture mediums to 5.0 × 10^5^ cells/mL. Cell size was measured before spreading on substrate by Image J. Diameters of Jurkat, PC 12 and MDCK cells were 9.8 ± 1.6, 11.8 ± 1.2 and 15.3 ± 1.7 µm, respectively. Before experiment, cell aggregates were removed by a 40-μm sterile strainer (EASYstrainer, Greiner Bio-One).

### Groove visualization

Scanning electron microscope (SEM, Hitachi SU-1510) was employed to visualize the cross-sections of the grooves engraved by fs laser at four different pulse energies of 0.7, 1.4, 2.1 and 2.8 µJ/pulse. The sample substrates were cut by a diamond knife to visualize the width and depth of the grooves at the cross-section. The SEM images were collected at 1.0 kV accelerated voltage. Five grooves were analyzed for each condition with data shown as mean ± standard deviation (SD).

### Groove roughness analysis

Atomic force microscopy (AFM, JPK instruments, NanoWizard 4) was used to measure the roughness of the surficial area between the neighboring two grooves [Fig. S1(a)]. QITM mode was conducted using a pyramidal silicon tip (HQ-XSC11/No Al, MicroMasch) with spring constant of 42 N/m, tip radius at 8 nm, half-cone angle at 20° and height at 15 μm. A 20 × 20-μm area was randomly selected to include the groove surrounding area [Fig. S1(b)]. A 5 × 5-μm area was imaged with a resolution of 10 nm. Within the imaged area, a 3 × 3-μm square close to the groove was analyzed. The collected height data were calibrated using the software JPK data processing 6.1 for the calculation of RMS roughness.

### Numerical simulation

The software COMSOL Multiphysics 5.4 was used to simulate the fluid behaviors induced by the open v-shaped microstructures within the microfluidic channel. Simplified 3D models were built based on the geometry information from SEM images. A microchannel without groove microstructures was established as a control. Fluid velocity fields were computed in the laminar flow regime under a stationary condition. Newtonian fluid water was selected for injection with an inlet fluid flow rate of 450 nL/min. Fluids flowing through the channel were visualized with streamlines to evaluate the fluidic behaviors induced by the grooves of different depths.

## Supplementary information

Supplementary Figures.

Supplementary Video S1.

Supplementary Video S2.

Supplementary Video S3.

Supplementary Video S4.

Supplementary Video S5.

## References

[1] Beebe DJ, Mensing GA, Walker GM (2002). Physics and Applications of Microfluidics in Biology. Annu. Rev. Biomed. Eng..

[2] Weibel DB, Whitesides GM (2006). Applications of microfluidics in chemical biology. Curr. Opin. Chem. Biol..

[3] Whitesides GM (2006). The origins and the future of microfluidics. Nature.

[4] Howell PB (2008). Two simple and rugged designs for creating microfluidic sheath flow. Lab. Chip.

[5] Karimi A, Yazdi S, Ardekani AM (2013). Hydrodynamic mechanisms of cell and particle trapping in microfluidics. Biomicrofluidics.

[6] Álvarez-Barrientos A, Arroyo J, Cantón R, Nombela C, Sánchez-Pérez M (2000). Applications of flow cytometry to clinical microbiology. Clin. Microbiol. Rev..

[7] Nitta N (2018). Intelligent image-activated cell sorting. Cell.

[8] Martel JM, Toner M (2014). Inertial focusing in microfluidics. Annu. Rev. Biomed. Eng..

[9] Kern W (2004). Determination of relapse risk based on assessment of minimal residual disease during complete remission by multiparameter flow cytometry in unselected patients with acute myeloid leukemia. Blood.

[10] Nitta N (2020). Raman image-activated cell sorting. Nat. Commun..

[11] Iino T (2019). High-speed microparticle isolation unlimited by Poisson statistics. Lab Chip.

[12] Li M, van Zee M, Goda K, Di Carlo D (2018). Size-based sorting of hydrogel droplets using inertial microfluidics. Lab. Chip.

[13] Zhang T (2020). Focusing of sub-micrometer particles in microfluidic devices. Lab. Chip.

[14] Wang R, Du J, Guo W, Zhu Z (2016). Investigation on the thermophoresis-coupled inertial sorting of submicrometer particles in a microchannel. Nanoscale Microscale Thermophys. Eng..

[15] Li M (2012). Continuous particle focusing in a waved microchannel using negative dc dielectrophoresis. J. Micromech. Microeng..

[16] Zhao Y, Fujimoto BS, Jeffries GD, Schiro PG, Chiu DT (2007). Optical gradient flow focusing. Opt. Exp..

[17] Augustsson P, Karlsen JT, Su HW, Bruus H, Voldman J (2016). Iso-acoustic focusing of cells for size-insensitive acousto-mechanical phenotyping. Nat. Commun..

[18] Wiklund M (2012). Acoustofluidics 12: Biocompatibility and cell viability in microfluidic acoustic resonators. Lab Chip.

[19] Xuan X, Zhu J, Church C (2010). Particle focusing in microfluidic devices. Microfluid. Nanofluid..

[20] Huang LR, Cox EC, Austin RH, Sturm JC (2004). Continuous particle separation through deterministic lateral displacement. Science.

[21] Vernekar R, Krüger T, Loutherback K, Morton K, Inglis DW (2017). Anisotropic permeability in deterministic lateral displacement arrays. Lab Chip.

[22] Zhang J (2016). Fundamentals and applications of inertial microfluidics: a review. Lab Chip.

[23] Di Carlo D (2009). Inertial microfluidics. Lab Chip.

[24] Freund JB (2014). Numerical simulation of flowing blood cells. Annu. Rev. Fluid Mech..

[25] Ji H, Sander D, Haas A, Abshire PA (2007). Contact imaging: simulation and experiment. IEEE Trans. Circuits Syst. I. Regul. Pap..

[26] Chen YC, Clegg RM (2009). Fluorescence lifetime-resolved imaging. Photosynth. Res..

[27] Kim JY, Ahn SW, Lee SS, Kim JM (2012). Lateral migration and focusing of colloidal particles and DNA molecules under viscoelastic flow. Lab Chip.

[28] D’Avino G (2012). Single line particle focusing induced by viscoelasticity of the suspending liquid: theory, experiments and simulations to design a micropipe flow-focuser. Lab Chip.

[29] Asghari M, Serhatlioglu M, Ortaç B, Solmaz ME, Elbuken C (2017). Sheathless microflow cytometry using viscoelastic fluids. Sci. Rep..

[30] Kang, K., Lee, S. S., Hyun, K., Lee, S. J. & Kim, J. M. DNA-based highly tunable particle focuser. *Nat. Commun.* **4**, (2013). 10.1038/ncomms3567 24108276

[31] Choi S, Song S, Choi C, Park JK (2009). Hydrophoretic sorting of micrometer and submicrometer particles using anisotropic microfluidic obstacles. Anal. Chem..

[32] Hsu CH, Di Carlo D, Chen C, Irimia D, Toner M (2008). Microvortex for focusing, guiding and sorting of particles. Lab Chip.

[33] Choi S, Park JK (2009). Optically coated mirror-embedded microchannel to measure hydrophoretic particle ordering in three dimensions. Small.

[34] Sugioka K, Cheng Y (2012). Femtosecond laser processing for optofluidic fabrication. Lab Chip.

[35] Sugioka K (2014). Femtosecond laser 3D micromachining: a powerful tool for the fabrication of microfluidic, optofluidic, and electrofluidic devices based on glass. Lab Chip.

[36] Ren K, Zhou J, Wu H (2013). Materials for microfluidic chip fabrication. Acc. Chem. Res..

[37] Krol DM (2008). Femtosecond laser modification of glass. J. Non. Cryst. Solids.

[38] Sundaram SK, Mazur E (2002). Inducing and probing non-thermal transitions in semiconductors using femtosecond laser pulses. Nat. Mater..

[39] Gattass RR, Mazur E (2008). Femtosecond laser micromachining in transparent materials. Nat. Photonics.

[40] Iliescu C, Jing J, Tay FEH, Miao J, Sun T (2005). Characterization of masking layers for deep wet etching of glass in an improved HF/HCl solution. Surf. Coatings Technol..

[41] Li X, Abe T, Esashi M (2001). Deep reactive ion etching of Pyrex glass using SF6 plasma. Sens. Actuators A-Phys..

[42] Shah L, Tawney J, Richardson M, Richardson K (2001). Femtosecond laser deep hole drilling of silicate glasses in air. Appl. Surf. Sci..

[43] Hnatovsky C (2006). Fabrication of microchannels in glass using focused femtosecond laser radiation and selective chemical etching. Appl. Phys. A Mater. Sci. Process..

[44] Matsuo S (2008). Laser microfabrication and rotation of ship-in-a-bottle optical rotators. Appl. Phys. Lett..

[45] Yalikun Y, Hosokawa Y, Iino T, Tanaka Y (2016). An all-glass 12 μm ultra-thin and flexible micro-fluidic chip fabricated by femtosecond laser processing. Lab Chip.

[46] Yalikun Y, Tanaka N, Hosokawa Y, Iino T, Tanaka Y (2016). Ultrathin glass filter fabricated by femtosecond laser processing for high-throughput microparticle filtering. Appl. Phys. Express.

[47] Wu RM, Lin MH, Lin HY, Hsu RY (2006). 3D simulations of hydrodynamic drag forces on two porous spheres moving along their centerline. J. Colloid Interface Sci..

[48] Oliveira MSN, Pinho FT, Alves MA (2012). Divergent streamlines and free vortices in Newtonian fluid flows in microfluidic flow-focusing devices. J. Fluid Mech..

[49] Hagiwara M, Kawahara T, Arai F (2012). Local streamline generation by mechanical oscillation in a microfluidic chip for noncontact cell manipulations. Appl. Phys. Lett..

[50] Chung AJ, Gossett DR, Di Carlo D (2013). Three dimensional, sheathless, and high-throughput microparticle inertial focusing through geometry-induced secondary flows. Small.

[51] van der Hoef MA, Beetstra R, Kuipers JAM (2005). Lattice-Boltzmann simulations of low-Reynolds-number flow past mono-and bidisperse arrays of spheres: results for the permeability and drag force. J. Fluid. Mech..

[52] Hosokawa Y (2019). Applications of the femtosecond laser-induced impulse to cell research. Jpn. J. Appl. Phys..

[53] Squires TM, Quake SR (2005). Microfluidics: Fluid physics at the nanoliter scale. Rev. Mod. Phys..

[54] Oikawa K (2015). Physical interaction between peroxisomes and chloroplasts elucidated by in situ laser analysis. Nat. Plants.

[55] Hosokawa Y, Hagiyama M, Iino T, Murakami Y, Ito A (2011). Noncontact estimation of intercellular breaking force using a femtosecond laser impulse quantified by atomic force microscopy. Proc. Natl. Acad. Sci. U. S. A..

[56] Loudet JC, Hanusse P, Poulin P (2004). Stokes drag on a sphere in a nematic liquid crystal. Science.

[57] Lee MG, Shin JH, Bae CY, Choi S, Park JK (2013). Label-free cancer cell separation from human whole blood using inertial microfluidics at low shear stress. Anal. Chem..

[58] McFaul SM, Lin BK, Ma H (2012). Cell separation based on size and deformability using microfluidic funnel ratchets. Lab Chip.

[59] Sugioka K, Cheng Y (2014). Femtosecond laser three-dimensional micro-and nanofabrication. Appl. Phys. Rev..

[60] Sima F (2018). Three-dimensional femtosecond laser processing for lab-on-a-chip applications. Nanophotonics.

