# Supplementary material for: Hydrodynamic particle focusing enhanced by femtosecond laser deep grooving at low Reynolds numbers
Source: Sci Rep. 2021 Jan 18;11:1652. doi: 10.1038/s41598-021-81190-y (PMC7813873; doi:10.1038/s41598-021-81190-y)
Supplement: Supplementary file 1 — Supplementary Figures. [file 41598_2021_81190_MOESM1_ESM.pdf]

Hydrodynamic particle focusing enhanced by femtosecond laser deep grooving at low Reynolds numbers

Tianlong Zhang[1,2], Misuzu Namoto[1], Kazunori Okano[1], Eri Akita[1], Norihiro Teranishi[1], Tao Tang[1], Dian Anggraini[1], Yansheng Hao[1], Yo Tanaka[3], David Inglis[2], Yaxiaer Yalikun[1]*, Ming Li[2]*, and Yoichiroh Hosokawa[1]

1. Division of materials science, Graduate School of Science and Technology, Nara Institute of Science and Technology, 630-0192, Ikoma, Japan
2. School of Engineering, Macquarie University, Sydney 2122, Australia
3. Center for Biosystems Dynamics Research, RIKEN, Osaka 565-0871, Japan

*Email: ming.li@mq.edu.au
yaxiaer@ms.naist.jp

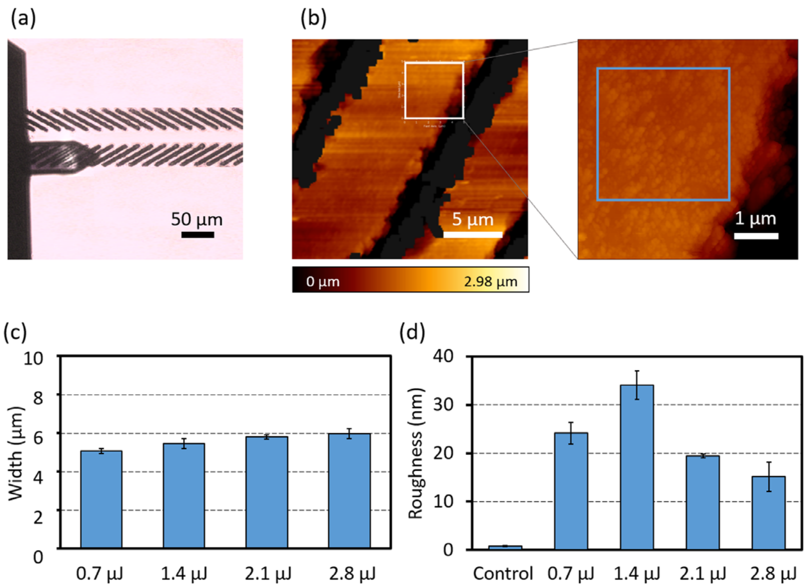

Figure S1 Laser engraved microstructures detected by atomic force microscopy (AFM). (a) AFM operation under optical microscope. (b) A 20 ×20-μm image showing the groove surrounding area. Zoom-in is a 5 ×5-μm image. The blue box within the image is a 3 ×3-μm area for roughness calculation. (We used the software JPK data processing 6.1 to create the image). (c) Groove widths achieved by four different pulse energies of 0.7, 1.4, 2.1 and 2.8 μJ/pulse. (d) Roughness for the control (plain glass substrate) and the laser engraved groove groups. N = 3 for each condition.

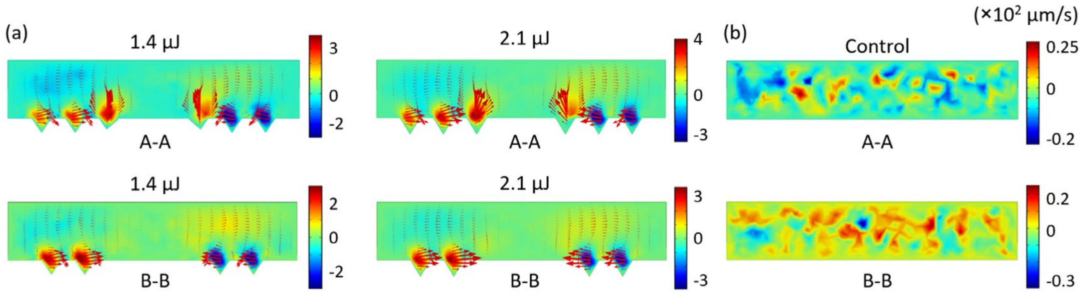

Figure S2 2D vector plots of flow velocity fields at cross sections of (a) the microchannel with four or six grooves when the pulse energies are 1.4 μJ/pulse and 2.1 μJ/pulse and (b) plain microchannel without grooves (control). Red arrows are scalars shown in a proportional way. (We used the software COMSOL Multiphysics 5.4 to create the image).

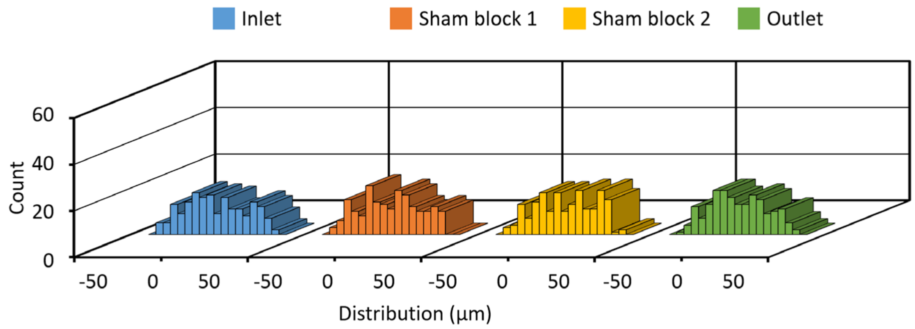

Figure S3 Plots of the lateral distributions of 10-μm polystyrene particles along channel width for the control groups (a channel without groove microstructures). The injection flow rate is 450 nL/min. Particle lateral positions were detected at four different locations: inlet (before passing sham block 1), after passing the sham block 1, 2, and 3 (outlet). N = 200 for each group.

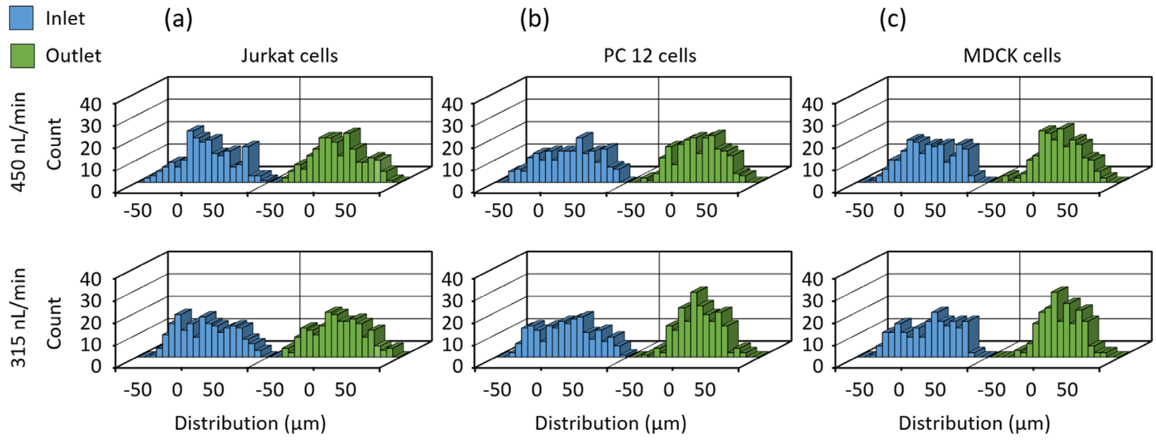

Figure S4 Focusing of live cells in the microfluidic device with fs laser patterned arrays of open v-shaped microstructures. The lateral positions of (a) Jurkat cells, (b) PC 12 cells and (c) MDCK cells at the inlet and outlet are recorded and compared. The flow rates are 315 and 450 nL/min. 200 cells are measured for each group.

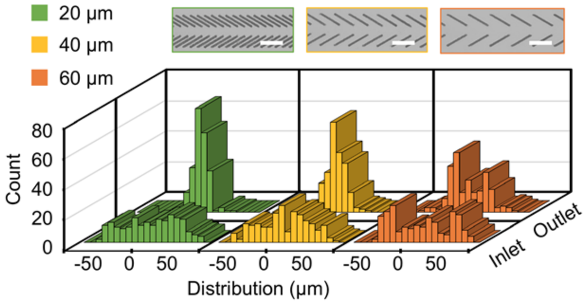

Figure S5 Effect of the interval distance between grooves on the focusing of 10-μm particles in the microchannel with four blocks. Particles are injected into the microchannel with an injection flow rate at around 450 nL/min.[1] The patterned grooves have three different distances: 20 μm, 40 μm, and 60 μm. Green denotes particle distributions at inlet (1.0 ± 22.5 μm) and outlet (0 ± 7.0 μm) for the channel with grooves at interval distance of 20 μm. Yellow denotes particle distributions at inlet (-0.2 ± 20.5 μm) and outlet (0.3 ± 8.3 μm) for the channel with grooves at interval distance of 40 μm. Orange denotes particle distributions at inlet (0.9 ± 23.7 μm) and outlet (-2.7 ± 15.4 μm) for the channel with grooves at interval distance of 60 μm. Optical images of these three types of grooves are presented. Scale bar is 60 μm. 200 particles are measured for each group.

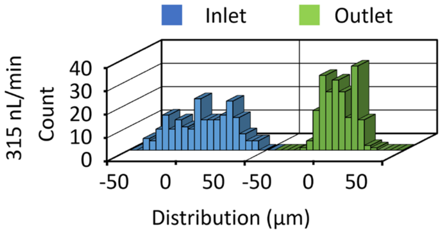

Figure S6 Distributions of 10-μm polystyrene particles at inlet (1.4 ± 21.9 μm) and the end of the first block (-0.6 ± 11.5 μm) with an injection flow rate at 315 nL/min.

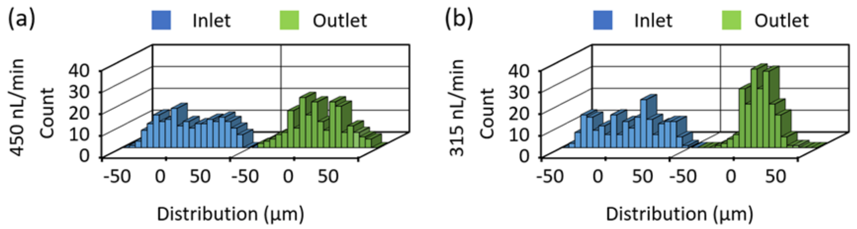

Figure S7 Distributions of 4.5-µm polystyrene particles. (a) Particle distributions at inlet (0.6 ± 24.1 µm) and the end of the third block (0 ± 20.5 µm) with an injection flow rate of 450 nL/min. (b) Particle distributions at inlet (0.1 ± 25.4 µm) and the end of the third block (1.2 ± 11.4 µm) with an injection flow rate of 315 nL/min.

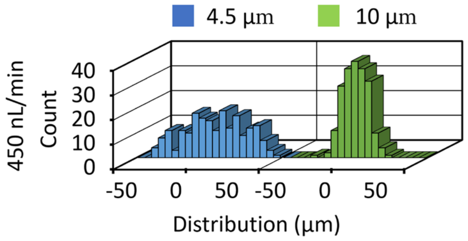

Figure S8 Distributions of 4.5-µm polystyrene particles (1.8 ± 22.2 µm) and 10-µm polystyrene particles (-1.8 ± 9.0 µm) at the end of the third block with an injection flow rate of the particle mixtures at 450 nL/min. The concentration of the injected 4.5- and 10-µm polystyrene particles is 2.5×10^5 particles/mL.

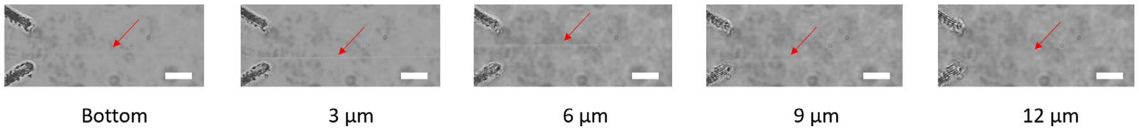

Figure S9 Images of particle traces at the end of the third block when the 60× objective lens is focused at different height levels. It is estimated that the distance between the center of the 10-µm particle and the channel bottom ranges from 5 to 7 µm. The red arrow denotes the particle trace. The injection flow rate is 450 nL/min. Scale bar is 20 µm.

Reference

[1]Huh, D. *et al.* Gravity-driven microfluidic particle sorting device with hydrodynamic separation amplification. *Anal. Chem.* **79**, 1369-1376 (2007).
